# The Impact of Lockdown on Couples’ Sex Lives

**DOI:** 10.3390/jcm10071414

**Published:** 2021-04-01

**Authors:** Elisabetta Costantini, Francesco Trama, Donata Villari, Serena Maruccia, Vincenzo Li Marzi, Franca Natale, Matteo Balzarro, Vito Mancini, Raffaele Balsamo, Francesco Marson, Marianna Bevacqua, Antonio Luigi Pastore, Enrico Ammirati, Marilena Gubbiotti, Maria Teresa Filocamo, Gaetano De Rienzo, Enrico Finazzi Agrò, Pietro Spatafora, Claudio Bisegna, Luca Gemma, Alessandro Giammò, Alessandro Zucchi, Stefano Brancorsini, Gennaro Ruggiero, Ester Illiano

**Affiliations:** 1 Department of Surgical and Biomedical Science, Andrological and Urogynecological Clinic, Santa Maria Terni Hospital, University of Perugia, 05100 Terni, Italy; elisabetta.costantini@unipg.it (E.C.); ester.illiano@inwind.it (E.I.); 2 Urology Clinic, Careggi Hospital, University of Florence, 50100 Florence, Italy; donata.villari@unifi.it (D.V.); limarzi2012@gmail.com (V.L.M.); pietro.spatafora@hotmail.it (P.S.); claudio.bisegna@gmail.com (C.B.); gemmaluca.dr@gmail.com (L.G.); 3 Istituti Clinici Zucchi, 20861 Brughiero, Italy; serena.maruccia@gmail.com; 4 Urogynecologic Unit, S. Carlo Hospital, 00100 Rome, Italy; francanatale3@gmail.com; 5 Urology Clinic, University of Verona, 37100 Verona, Italy; matteo.balzarro@aovr.veneto.it; 6 Urology Clinic, University of Foggia, 71100 Foggia, Italy; mancini.uro@gmail.com; 7 Urology Clinic, Monaldi Hospital, 80125 Naples, Italy; r.bals@virgilio.it; 8 Urology Clinic, Chieri Carmagnola and Moncalieri Hospital, 10121 Turin, Italy; francescomarson84@gmail.com; 9 Urology Clinic, Bianchi-Malacrino-Morelli Hospital, 89121 Reggio Calabria, Italy; mariannachiarabevacqua@gmail.com; 10 Urology Clinic, ICOT, Department of Medical Surgical Sciences and Biotechnologies, Sapienza University of Rome, 04100 Latina, Italy; antopast@hotmail.com; 11 Urology Clinic, Città della Salute e della Scienza, 10121 Turin, Italy; ammirati.enrico@gmail.com (E.A.); giammo.alessandro@gmail.com (A.G.); 12 Urology Clinic, San Donato Arezzo Hospital, 52100 Arezzo, Italy; marilena.gubbiotti@gmail.com; 13 Urology Clinic, ASL CN1, 12038 Savigliano, Italy; mt.fil@libero.it; 14 Urology Clinic, University of Bari, 70121 Bari, Italy; gaetanoderienzo@gmail.com; 15 Urology Clinic, Torvergata University, 00100 Rome, Italy; finazzi.agro@med.uniroma2.it; 16 Urology clinic, University of Pisa, 56121 Pisa, Italy; zucchi.urologia@gmail.com; 17 Department of Experimental Medicine, University of Perugia, 05100 Terni, Italy; stefano.brancorsini@unipg.it; 18 Department of Psychology, Laboratory of Cognitive Science and Immersive Virtual Reality, Univerity of Campania, Luigi Vanvitelli, 81100 Caserta, Italy; gennaro.ruggiero@unicampania.it

**Keywords:** Covid-19, lockdown, male sexual dysfunction, female sexual dysfunction

## Abstract

Background: the aim of this study was to perform an Italian telematics survey analysis on the changes in couples’ sex lives during the coronavirus disease 2019 (COVID-19) lockdown. Methods: a multicenter cross sectional study was conducted on people sexually active and in stable relationships for at least 6 months. To evaluate male and female sexual dysfunctions, we used the international index of erectile function (IIEF-15) and the female sexual function index (FSFI), respectively; marital quality and stability were evaluated by the marital adjustment test (items 10–15); to evaluate the severity of anxiety symptoms, we used the Hamilton Anxiety Rating Scale. The effects of the quarantine on couples’ relationships was assessed with questions created in-house. Results: we included 2149 participants. The sex lives improved for 49% of participants, particularly those in cohabitation; for 29% it deteriorated, while for 22% of participants it did not change. Women who responded that their sex lives deteriorated had no sexual dysfunction, but they had anxiety, tension, fear, and insomnia. Contrarily, men who reported deteriorating sex lives had erectile dysfunctions and orgasmic disorders. In both genders, being unemployed or smart working, or having sons were risk factors for worsening the couples’ sex lives. Conclusion: this study should encourage evaluation of the long-term effects of COVID-19 on the sex lives of couples.

## 1. Introduction

On 21 February, 2020, in Italy, the first cases of severe acute respiratory syndrome coronavirus 2 (SARS-CoV-2)—the coronavirus responsible for coronavirus disease 2019 (COVID-19)—were documented. The number of cases quickly increased, leading to a pandemic. On 10 March, 2021, a total of 487,074 cases and 100,811 deaths were reported in Italy [1].

On 5 March, 2020, a national lockdown was declared (Phase 1). For 50 days, this lockdown affected all national production sectors and health services; non-urgent ambulatorial [2] and surgical activities [3] were suspended in all Italian hospitals.

The restrictions prevented families, friends, and sometimes non-cohabiting couples from physically meeting. On 4 May, 2020 “Phase 2” began, which allowed people to meet family members and relatives living in the same city, but other restrictions were unchanged. The lockdown has impacted the entire population; people of all age groups have changed their habits, which has led to increased uncertainty about the future, especially in regards to (often irreversible) changes, such as job loss.

In Italy, according to the National Institute of Statistics (ISTAT) [4], after the substantial stagnation of the first two months in 2020 (−0.1% in January and +0.1% in February), the onset of the pandemic hit the work market, causing a reduction of 124,000 employees (−0.5%) in March, more than double that number in April (−274 thousand, −1.2%), and a continuation in May (−84 thousand, −0.4%). The job market and financial insecurity were related to symptoms of depression and anxiety [5,6]. Repercussions of the lockdown on the Italian population’s sex lives are less known. In Italy, crises that have changed peoples’ habits (e.g., the L’Aquila earthquake) have been studied. It was shown that crises can alienate loved ones; moreover, the loss of a home can alter daily habits and couples’ sex lives. After the earthquake, there was a high rate of sexual dysfunction-related symptoms in young adults, particularly subjects experiencing post-traumatic stress disorder [7]. The lockdown has also likely led to changes in the sex lives of Italians.

Overall, evidence of the impacts of external stressful events on couples’ sex lives is still being debated. Although a few studies have addressed the issue of sexual behavior during the pandemic [8,9,10,11,12], to our knowledge, there is not much data [13] on Italian couples’ sex lives during events such as the lockdown that also investigated sexual activity and functioning. Since intimate relationships can be a reflection of the “goodness” of couples’ psychological and physical states, we investigated if (and to what extent) uncertainty and perceived danger could, albeit temporarily, cause changes in the sex lives of Italian couples. Studying these factors could allow us to better understand the effects of social deprivation and of perceived/actual danger, as well as how couples are able to compensate for a long-term lack of basic psychological needs.

We hypothesize that the lockdown influenced the couples’ sex lives. The aim of this study was to perform a telematics survey analysis of the changes in the sexual behavior of adult men and women in stable relationships during Phase 1 of the lockdown.

## 2. Methods

This was an Italian, multicenter cross-sectional study, conducted from 15 urological centers. It was approved by the local ethics committee, and participants signed online informed consent documents.

### 2.1. Participants

The study was conducted from 4 May 2020 (50 days after the start of the lockdown) to 18 May 2020. Inclusion criteria were: subjects sexually active, in stable relationships for at least 6 months, both sexes, and of any age. Exclusion criteria were: subjects who were COVID-19 positive, single, or sexually inactive.

### 2.2. Procedure

The research was performed with an online survey. A questionnaire was created in Italian via Google Forms (Supplementary Material 1). The questionnaire link was forwarded to all investigator associates. Respondents were recruited through convenience sampling and were asked to forward (or post) the links among their contact groups in all social networks (i.e., Facebook) or by free communication apps (i.e., WhatsApp).

Clicking on the questionnaire link caused the consent form and the instructions to appear on the screen. The questionnaire became accessible after participants accepted the terms and conditions of the study. Data cleaning was performed by one of the investigators and was cross-checked by a second investigator.

### 2.3. Measures

For each participant, demographic data were obtained, including age, gender, weight, and height, as freeform questions; and sexual orientation, current region of residence, and occupation as multiple choice questions. Other questions were: “Do you have children?” and “Do you live with your partner?” with dichotomous answers; and “How many years have you been in a relationship with your partner?”.

In regards to sexual functioning, women had to fill in the female sexual function index (FSFI-19 items) [14], and the international index of erectile function (IIEF-15 items) [15] was used to evaluate female and male sexual function in the 4 weeks before the survey, during Phase 1. Married participants completed the marital adjustment test (MAT), items 10 to 15 [16], which evaluated marital quality and stability. The items chosen investigated couple complicity and the presence of common interests

All participants completed the Hamilton Anxiety Rating Scale (HAM) [17], which measures the severity of anxiety symptoms. The effects of the COVID-19 pandemic and quarantine on couples’ relationships were assessed with questions created in-house (Table 1). We defined “improvement of sexual life” with the answer “Much; very much” to Item 4 (“Do you think that your couple’s sex life has improved during this period?”) of COVID-19; while “worsening of sexual life” with the answer “Much; very much” to Item 3 (“Do you think that your sex life as a couple has deteriorated during this period?”) of COVID-19.

### 2.4. Statistical Analysis

All answers were downloaded via Google Form and reported in a calculation file, and each answer was converted into a corresponding score on the basis of the questionnaire analyzed. The Mann–Whitney test was used to compare ordinal and non-normally distributed continuous variables; to compare normally distributed continuous variables we used T-Test. Categorical data were analyzed with the χ2 test with Yates correction or Fisher’s exact test. Multivariate logistic regression models were fit for the prediction of risk factors (clinical and demographic data) for worsening female and male sex lives. Multivariate logistic regression models were fit, incorporating all variable analyses in bivariate analyses. Odds ratios (ORs) with 95% CIs were also calculated. Statistical analyses were performed in software (SPSS, Version 23.0; IBM Corp, Armonk, NY, USA). A two-sided *p* value < 0.05 was considered significant. We did not perform the correction for multiplicity. The internal validity of the COVID-19 questionnaire was evaluated by Cronbach alpha test.

## 3. Results

From 4 May to 18 May 2020, we enrolled 2150 participants. One participant was excluded because the questionnaire had been filled out incorrectly. The analysis was performed on 2149 participants. Table 2 shows the demographic data of the study’s total population and both genders. The sample size for homosexuals and bisexuals was too small to perform statistical analysis; therefore, the analysis was performed on the entire population regardless of sexual orientation. The COVID-19 questionnaire showed a high level of inter-item reliability and Cronbach’s alpha (0.76).

A total of 49% replied “much or very much” to the question “Do you think that your couple’s sex life has improved during this period?” (Item 4 COVID-19); 29% replied “much or very much” to the question “Do you think that your couple’s sex life has deteriorated during this period?” (Item 3 COVID-19); and 22% did not report a change—subjects who answered that they neither had an improvement nor a worsening (Items 3 and 4, COVID-19).

Table 3 shows the demographic data of the general population who reported an improvement or worsening, or no changes of couples’ sex lives.

Participants who reported improved sex lives were mostly female (*p* = 0.01) or participants with lasting relationships (*p* < 0.0001), compared to those who reported deteriorating sex lives, or having no changes during the lockdown.

Subjects who reported worsening sex lives were unmarried (*p* = 0.01) or had sons (*p* < 0.0001) compared to other groups.

Male subjects (*p* = 0.01), participants with shorter relationships (*p* < 0.0001), or participants without sons (*p* < 0.0001) reported no changes compared to participants in others groups.

There was no statistical difference according to age.

### 3.1. The Improvement of Couples’ Sex Lives

Improvements in couples’ sex lives was reported more by female subjects than male (*p* = 0.002); improvements were also associated with the following variables: cohabiting with the partner (84%), being in a stable relationship for more than 5 years (72.8%), married, without sons (59.7%). Both men and women who reported an improvement in their sex lives (Item 4 COVID-19) had good relationships with their partners.

Among women, 97% of them had all (or some) interests in common with their partners (Item 2 MAT (Marital Adjustment Test)), and 65.7% liked to do the same activities as their partners in their free time (they gave the same answers to Items 4–5 in the MAT), and 70% would remarry the same person (Item 6 MAT).

Among men, 72% and 74% were happy (Item 7 COVID-19) and satisfied with their partners (Item 10 COVID-19), respectively; 83% had all or some interests in common with their partners (Item 2 MAT), and 66% liked to do the same activities as their partners in their free time (Items 4–5 MAT). A total of 80% would remarry the same person (Item 7 MAT).

None of the subjects, in either gender, had sexual dysfunctions. Men experienced good erectile and orgasmic functions, high sexual desire, and overall satisfaction (IIEF mean 33.5 ± 21.4) in the prior 4 weeks, while women had a median FSFI score of 28.5 (2–35.6). They lived mostly in central (39.3%) or south (30.9%) Italy; most attended graduate school (94%) and worked at the usual workplaces (61.9%).

### 3.2. Worsening of Couples’ Sex Lives

Worsening of couples’ sex lives was reported in both sexes, but without statistically significant differences between genders (*p* = 0.4) or between people under and over 40 years of age (47.4% vs. 48.6%, *p* = 0.8). Most of these subjects did not live with their partners during lockdown (73.4%). Among those who reported worsening sex lives (as a couple) and who lived with their partners (26.6%), 82% had sons, and 81.7% had stable relationships for more than 5 years. In 50.8% of the cases, they lived in north Italy; most attended high school (55.7%), and worked at home in smart working (68.7%).

Women who responded that “their couple’s sex lives has deteriorated” (Item 3 COVID-19) had no sexual dysfunction (FSFI median score 28.5 (2–35.6)) (Table 4). However, they had higher anxiety (17.7% vs. 8.4%, *p* < 0.0001, item 1 HAM score), tension (21.6% versus 10.5%, *p* < 0.0001, Item 2 HAM score), fear (21.3% vs. 7.2% *p* < 0.0001, Item 3 HAM score), and insomnia (27.5% vs. 3.7%, *p* < 0.0001, Item 4 HAM score) than women who had replied “no, not much or so-so” to the question of whether their sex life had worsened. In addition, among cohabitants, women were also more likely to be dissatisfied with their partners (13.8% vs. 5.3%, *p* < 0.0001) and to feel nervous toward their partners during this period (24.6% vs. 5.1%, *p* < 0.0001) than women who had replied “no, not much or so-so” to the question on whether their sex life had worsened.

In contrast, men who reported worsened couples’ sex lives had mild erectile dysfunctions, orgasmic dysfunctions, and low sexual satisfaction in the prior 4 weeks (Table 4). These results were conformed in the univariate and multivariate analysis (Table 5); in fact, erectile dysfunction, orgasmic dysfunction, and low intercourse satisfaction were risk factors for worsened couples’ sex lives. Cohabitating men (57%) had mild erectile dysfunction (19 ± 10.5), pathological scores of desire (of 5.5 ± 2.1), and overall satisfaction of 5.5 ± 2.1. They felt more uncomfortable with their partners at home than those who did not report worsened sex lives (12.9% vs. 5.4%, *p* < 0.0001, item 8 COVID-19), and had mild anxiety symptoms (median score 17 (8–32)). However, only 11.3% of married people said that they would marry another person (Item 6 MAT Test), 19.5% experienced an increase in couples’ problems (Item 13 COVID-19), and 7% were unhappy with their partners at home (Item 12 COVID-19). Among non-cohabitants the IIEF scores, except for sexual desire (Table 4) and moderate symptoms of anxiety (median score 29 (8–35)), were associated with a worsening of couples’ sex lives.

Table 6 and Table 7 showed the results of univariate analysis and multivariate logistic regression for risk factor assessment for worsening female and male sex lives, respectively. In both genders, being unemployed or smart working during this period, as well as living in north Italy, having sons, and a relationship for more than 5 years, were risk factors for worsened sex lives.

### 3.3. Risk Factors of Worsening of Couples’ Sex Lives

In the univariate, being married was a risk factor for both women and men, but the result was not confirmed at the multivariate. Anxiety, fear, and insomnia that developed during this period appeared to be risk factors for worsened sex lives in both genders.

The same risk factors were obtained in the univariate and multivariate logistic regression for risk factor assessment for the worsened sex lives of female and male cohabitants.

A total of 76% of the population replied, “no or not much” to the question “Do you feel safe outside your home?” (Item 2 COVID-19). According to the HAM scores, participants feeling insecure while away from home had higher anxiety (88.2% vs. 11.5%, *p* = 0.002), tension, fatigue, alarm responses, crying, trembling, restlessness, inability to relax (90.7% vs. 9.3%, *p* = 0.04), fear (99.6% vs. 0.4% *p* < 0.0001), and insomnia (86.9% vs. 13.1%, *p* = 0.04) than participants who replied “much or very much.”

A total of 90% of the respondents replied that “they felt very or very safe inside the home” (Item 1 COVID-19), and 84.5% that “they felt safe at home with their partner” (Item 5 COVID-19).

## 4. Discussion

The combination of isolation and risk of contagion has provoked a negative cumulative effect in terms of psychological and socio-cognitive resilience. Isolation is a slow stress factor because it prevents social relationships that, in turn, help people regulate their emotions, cope with stressful events, and strengthen their resilience during difficulties [18,19]. Consistently, in the current study, as in an another Chinese cross-sectional survey [10], the majority of participants (especially those who reported feeling insecure outside the home) reported high levels of anxiety, fear, agitation, feelings of restlessness, and insomnia.

Our study showed that the COVID-19 pandemic has also influenced Italian couples’ sex lives. Simultaneously, the “lockdown” led to an improvement in couples’ sex lives in 49% of participants, particularly cohabitants, whereas 29% reported a worsened sex life for different reasons between men and women, and 22% reported no change.

During the lockdown, despite the impossibility of meeting friends and relatives, and maintaining stability, in many cases, a rapprochement occurred among cohabiting couples. Most people reported that they were satisfied and happy with their partners at home. The improvement was reported primarily in participants who had been in stable relationships for more than 5 years, probably because the increased time spent together favored the rediscovery of a feeling that the couple might have lost in their life routines. Spending entire days at home can stimulate and facilitate common interests between partners—the sharing of hobbies or daily practices that normally could not be shared because of a lack of time.

Participants over the age of 40 improved more than those younger than 40, probably because most of the younger participants did not live with their partners during this period.

In our study, in both genders, participants who reported an improvement in their sex lives did not have sexual dysfunction. It allowed them to obtain qualitatively valid sexual activity, which strengthened the couples, as such. Moreover, another Italian survey conducted on participants younger than those in the present study, showed improvements in the frequency of sexual intercourse and sexual desire in both genders [13]. Villani showed that the increase in frequency of sexual intercourse also caused an increase in spontaneous pregnancy during the lockdown [20].

The worsening of couples’ sex lives was reported in both genders, without a statistically significant difference between them (women 50.4% versus men 49.6%). In both genders, risk factors included being unemployed or smart working during this period, as well as living in north and central Italy, having sons, and a relationship for more than 5 years. The absence of children also likely allowed more time for couples. The partners were thus able to act on their sexual interests at any time. In everyday life, children attend school and, at times, are cared for by their grandparents after-school, and boys often engage in activities that allow parents to keep their space. However, the pandemic has changed the status quo, forcing couples to find fleeting moments of intimacy, which may not always be possible. During the pandemic period, couples with children were engaged in childcare and distance learning, and this negatively influenced the time available to devote to their partners and married lives.

Our study did not investigate the presence, during the lockdown, of other family members besides children; of course, this could also affect a couple’s sex life. However, we assumed that the decree allowed them to live only between cohabitants, and consequently, any other family members were present even before the lockdown; therefore, there should be no such influence to be analyzed.

Women who reporting a worsening of the couple’s sex life had no pathological median FSFI score, but they had emotional difficulties. Presumably, the worsening of women’s sex lives can be attributed to several factors, including the lockdown, psychological and sociocultural factors, and interpersonal well-being [21,22,23]. The pandemic, similar to other catastrophic situations (such as earthquakes, hurricanes or wars), could in fact cause anxiety and depression, thus, decrease the frequency of sexual intercourse [24], sexual desire [25], libido, orgasm, and vaginal lubrication [26,27].

In men, worsened sex lives were predominately due to sexual dysfunction; however, a low percentage of men were unhappy at home with their partners, and would marry other people. In this frame, presumably, we cannot exclude that, along with the lockdown effect, the link between sexual dysfunction and psychological well-being [28], cognitive attributions, depressive–anxiety state, partner reactions, automatic failure/disengagement thoughts, and ineffective coping styles may have played a critical role [29].

Another Italian survey showed a decrease in the number of times sexual intercourse took place during quarantine, compared with before the period, in particular the surveyed people who had sexual intercourse more than twice a week (54.2 vs. 37.2%, *p* < 0.01) [30]. It was due to lack of privacy (43.2%) and lack of stimuli (40.9%) [30].

During a pandemic, many problems are faced by non-cohabitant partners.

Lopes showed that, in cohabiting couples, an improvement of the couple’s sex life could be explained by the search for safety, intimacy, or by increasing the possibility of sexual intercourse [31]. In other cases, a worsening could be explained by opposite habits, and the search for compromises in respect of privacy and individuality [31]. The routine that was once taken for granted could become a source of stress. In these cases, sexual activity was certainly overshadowed. In unstable scenarios, men’s libidos can be affected as much as women; desire may increase (as a relief valve, to seek immediate pleasure) or may be completely absent (loss of sense of security and stability). While partners who do not live together can adopt new sexual routines [31]. Furthermore, other studies have showed that external stressful events can provoke a decrease in sexual activity and satisfaction [30,31].

Deprivation of sexual activity could have very insidious psychological effects, particularly at a time when people are fragile, and their mental health is particularly strained [31]. Often, the participants reported being alone at home, away from their partners, and the rest of their families. Each participant faced the sad situation in a different way, particularly from a sexual standpoint. In literature, for example, the impact of social distancing on sexuality has been evaluated in men undergoing radical prostatectomy [32]. It is a surgical procedure that has a high impact on male sexuality. Depression, anxiety, and deprivation of sexual activity, during lockdown, can decrease the desire for sexual rehabilitation; the subject then enters a vicious circle in which his emotions are a cause and effect of his sexual problems.

Previous studies have evaluated and even recommended the use of alternative sexual practices, such as masturbation [13] or “virtual sex” via digital platforms, such as phone or video chat [31] or viewing pornography movies [13]. One Italian survey reported decreased masturbation activity due to poor privacy (46.4%) and lack of desire (34.7%) [30]

The limitations of our study include the lack of baseline data on sexual dysfunction, although this aspect was not the aim of this study. Furthermore, considering the lack of information about any comorbidities, it is not possible to adjust results obtained for potential factors affecting sexual habits.

Other limitations include the sample being very large; however, it may not be representative of the general population. We have not analyzed data on homosexuals and bisexuals due to too small a sample; we used only four items of MAT questionnaires, and we could not include subjects who did not have the internet. Future studies should consider this.

The strengths of our study include the sample size. To our knowledge, the present study is the first to evaluate the relationship between the COVID-19 pandemic and Italian female and male sexual functions by using standardized questionnaires, evaluating sexual dysfunctions, and analyzing people in this mean age (43.07 ± 12.5).

## 5. Conclusions

In conclusion, the lockdown and social distancing during the COVID-19 pandemic mostly improved couples’ sex lives among cohabiting participants. The results of this research could be useful for interventions designed to help couples maintain sexual intimacy when they are not forced to spend more time together.

## Figures and Tables

**Table 1:** Questions concerning the coronavirus disease 2019 (COVID-19) pandemic and its effect on couples’ relationships.

	No	Not Much	So and So	Much	Very Much
1. Do you feel safe at home?					
2. Do you feel safe outside the home?					
3. Do you think that your sex life as a couple has deteriorated during this period?					
4. Do you think that your sex life as a couple has improved during this period?					
5. Do you feel safe with your partner at home?					
6. Do you feel dissatisfied with your partner at home?					
7. Do you feel happy with your partner at home?					
8. Do you feel uncomfortable with your partner at home?					
9. How comfortable do you feel with your partner at home?					
10. How satisfied do you feel with your partner at home?					
11. Do you think that your couple problems have decreased during this period?					
12. Do you feel unhappy with your partner at home?					
13. Do you think that your couple problems have increased during this period?					
14. Do you feel more nervous towards your partner during this period?					
15. Do you feel more calm towards your partner during this period?					

**Table 2:** The demographic data of the general population and female and male participants.

Data	Total Population *n* (2149)	Female *n* (1112)	Male *n* (1037)
Age (mean ± SD)	43.07 ± 12.5	43 ± 12.5	43.2 ± 12.4
BMI (median, range)	24.16 (18.90–44.2)	24.14 (18.90–43.1)	24.21 (19.90–44.2)
Sexual Orientation			
Heterosexual *n* (%)	2035 (94)	1075 (96.6)	960 (92.5)
Homosexual *n* (%)	91 (4)	18 (16.2)	73 (7)
Bisexual *n* (%)	23 (10)	19 (17)	4 (0.4)
Son *n* (%)	1253 (58)	657 (59)	596 (57.4)
Residences			
North *n* (%)	665 (31)	359 (32.2)	306 (29)
Central *n* (%)	773 (36)	417 (37.5)	356 (34.3)
South and Islands *n* (%)	711 (33)	336(30.3)	375 (36.7)
Education			
Primary school *n* (%)	7 (0.3)	2 (0.1)	5 (0.4)
Secondary school *n* (%)	132 (6)	78 (7)	54 (5.2)
High school *n* (%)	712 (33)	352 (31.7)	300 (28.9)
Graduate school *n* (%)	1298 (60)	680 (61.2)	678 (65.3)
Occupation			
Student *n* (%)	105 (4)	46 (4)	59 (5.7)
Retired *n* (%)	112 (5.2)	50 (4.5)	62 (5.9)
Unemployed *n* (%)	181 (8.4)	127 (11.4)	54 (5.3)
Working at the usual workplace *n* (%)	735 (3.4)	441 (39.6)	294 (28.4)
Smart working *n* (%)	1016 (4.7)	448 (40.3)	568 (54.7)
Cohabitants *n* (%)	1667 (77.5)	895 (80)	772 (74.4)
Married	1238 (5.7)		
Years of stable relationships			
≤1 years *n* (%)	171 (7.9)	162 (14.5)	109 (10.5)
Da 1 a 3 years *n* (%)	282 (13)	146 (1.43)	136 (13.2)
Da 3 a 5 years *n* (%)	204 (9.4)	130 (1.16)	74 (7.1)
≥5 years *n* (%)	1492 (69.4)	774 (69.6)	718 (69.2)
Questionnaire			
IIEF (mean ± SD)	38.9 ± 28.2	45.9 ± 12.2	34.2 ± 13.5
FSFI (median, range)	28.5 (2–35.6)	28.5 (2–35.6)	28.5 (2–35.6)
MAT (mean ± SD)	50.3 ± 2.6	47.3 ± 6.2	49.7 ± 3.5
HAM (mean ± SD)	5.1 ± 1.3	6.3 ± 2.5	5.3 ± 3.8

IIEF: International index of erectile function; FSFI: female sexual function index MAT: Marital adjustment test;HAM: Hamilton Anxiety Rating Scale.

**Table 3:** Demographic data on subjects who reported an improvement, worsening, or no changes in the couple’s sex life.

Data	Total Improvement	*p* Value	Total Worsening	*p* Value	Total No Change *n* = 477	*p* Value
	*n* = 1049		*n* = 623			
Age		0.2		0.4		0.4
>40 years (mean ± SD)	55.12 ± 3.2		57.25 ± 2.1		51.19 ± 3.8	
<40 years (mean ± SD)	34.6 ± 4.1		37.4 ± 2.6		31.7 ± 2.4	
BMI	23.5 ± 1.2		25.4 ± 2.9		26.4 ± 3.6	
Gender		0.002		0.4		0.4
Female *n* (%)	579 (55.2)		314 (50.4)		219 (45.9)	
Male *n* (%)	470 (44.8)		309 (49.5)		258 (54.0)	
Years of stable relationships		<0.0001		<0.0001		0.0001
≥5 year	764 (72.8)		399 (64.0)		148 (31.0)	
<5 years	285 (27.1)		224 (35.9)		329 (68.9)	
Married		0.001		<0.0001		<0.0001
Yes *n* (%)	640 (61)		306 (49.1)		292 (61.2)	
No *n* (%)	409 (38.9)		317 (50.8)		185 (38.7)	
Sexual Orientation		<0.0001		<0.0001		<0.0001
Heterosexual *n* (%)	985 (93.8)		580 (93)		470 (98.5)	
Homosexual *n* (%)	49 (4.6)		35 (5.6)		7 (1.4)	
Bisexual *n* (%)	15 (1.4)		8 (1.2)		0	
Educational		<0.0001		0.001		<0.0001
Primary school *n* (%)	0		7 (0.12)		0	
Secondary school *n* (%)	54 (5.14)		78 (12.5)		0 (0)	
High school *n* (%)	329 (31.3)		383 (61.4)		20 (4.1)	
Graduate school *n* (%)	985 (94)		313 (50.2)		457 (95.8)	
Residence		0.007		<0.0001		0.01
North *n* (%)	313 (29.8)		288 (46.2)		64 (13.4)	
South *n* (%)	324 (30.9)		218 (35.0)		231 (48.2)	
Centre *n* (%)	412 (39.3)		117 (28.4)		182 (38.1)	
Son		0.112		0.003		<0.0001
Yes *n* (%)	423 (40.3)		334 (53.6)		30 (6.2)	
No *n* (%)	626 (59.7)		289 (46.4)		447 (93.7)	
Occupation		0.001		<0.0001		<0.0001
Student *n* (%)	50 (0.2)		80 (12.8)		0	
Retired *n* (%)	85 (8.1)		10 (1.6)		17 (3.5)	
Unemployed *n* (%)	20 (2)		90 (14.4)		86 (18)	
Working at the usual workplace *n* (%)	650 (61.9)		20 (3.2)		65 (13.6)	
Smart working *n* (%)	244 (23.2)		423 (67.8)		309 (64.7)	
Questionnaire						
IIEF (mean ± SD)	37.4 ± 26.3		27 ± 28		32.6 ± 12	
FSFI (median, range)	28.5 (2–35.6)		28.5 (2–35.6)		28.5 (2.35–6)	
MAT (mean ± SD)	64.3 ± 2.1		42.7 ± 5.4		48.2 ± 2.6	
HAM (mean ± SD)	5.2 ± 3.5		9.7 ± 5.1		5.1 ± 2.3	

**Table 4:** Demographic data on subjects who lived with their partners during the lockdown, and reported an improvement, worsening, or no changes in the couple’s sex life.

Data	Total Improvement; *n* = 883	*p* Value	Total Worsening; *n* = 393	*p* Value	Total No Changes *n* = 389	*p* Value
Age		0.2		0.3		0.3
>40 years	54.09 ± 3.1		53.39±2.3		56.23 ± 3.4	
<40 years	36.4 ± 3.6		35.3±3.6		34.2 ± 2.7	
BMI	24.7± 2.9		26.3±3.6		27.4±3.1	
Gender		0.001		0.002		0.01
Female *n* (%)	491 (55.6)		217 (55.2)		200 (51.4)	
Male *n* (%)	392 (44.4)		176 (44.8)		189 (48.5)	
Years of stable relationships		<0.0001		<0.0001		<0.0001
≥5 year	721 (81.7)		321 (81.7)		310 (79.6)	
<5 years	162 (18.3)		721(18.3)		79 (20.3)	
Married		<0.0001		<0.0001		<0.0001
Yes *n* (%)	628 (71.1)		263 (66.9)		256 (65.8)	
No *n* (%)	255 (28.8)		130 (33.0)		133 (34.1)	
Educational		<0.0001		<0.0001		0.001
Primary school *n* (%)	0		4 (1.01)		2 (0.5)	
Secondary school *n* (%)	53 (6)		70(17.8)		189 (48.5)	
High school *n* (%)	300 (33.9)		219(55.7)		98 (25.1)	
Graduate school *n* (%)	530 (60.1)		100(25.4)		100 (25.7)	
Residence		0.005		0.001		<0.0001
North *n* (%)	229 (25.9)		200 (50.8)		75 (19.2)	
South *n* (%)	254 (28.7)		100 (25.4)		130 (33.4)	
Centre *n* (%)	400 (45.3)		3 (23.6)		184 (47.3)	
Son		0.001		<0.0001		<0.0001
Yes *n* (%)	400 (45.3)		325 (82)		135 (34.7)	
No *n* (%)	483 (54.7)		68 (17.3)		254 (65.3)	
Occupation		0.001		0.001		0.01
Student *n* (%)	0 (0.2)		0 (12.8)		125 (32.1)	
Retired *n* (%)	100 (11.3)		3 (0.7)		112 (28.7)	
Unemployed *n* (%)	13 (1.4)		100 (25.4)		0	
Working at the usual workplace *n* (%)	670 (75.8)		20 (5.1)		100 (25.7)	
Smart working *n* (%)	100 (11.3)		270(68.7)		25 (6.4)	
Sexual Orientation		<0.0001		<0.0001		<0.0001
Heterosexual *n* (%)	875 (93.8)		384 (97.7)		388(99.7)	
Homosexual *n* (%)	8 (0.9)		0		1 (0.25)	
Bisexual *n* (%)	0		9 (2.3)		0	
Questionnaire						
IIEF (mean ± SD)	33.5 ± 21.4		25 ± 12		34.8 ± 10.2	
FSFI (median, range)	28.5 (2–35.6)		28.5 (2–35.6)		28.5 (2.35–6)	
MAT (mean ± SD)	75.3 ± 3.5		41.7 ± 6.4		46.2 ± 2.4	
HAM (mean ± SD)	5.1 ± 2.4		9.2 ± 4.3		5.1 ± 2.7	

**Table 5:** Univariate analysis and multivariate logistic regression final model for female worsening sex lives vs. demographic and psychological data.

Female Worsening Sexual Life	Univariate Analysis		Logistic Regression	
	*p* Value	OR (95% CI)	*p* Value	OR (95% CI)
Age	0.63	0.56 (0.71–1.22)	0.98	0.98 (0.87–1.15)
BMI	0.77	0.66 (0.50–1.89)	0.51	0.66 (0.50–1.89)
Sexual Orientation				
Heterosexual	0.13	1.71 (0.74–3.94)	0.24	1.96 (0.62–6.15)
Homosexual	0.2	0.50 (0.14–1.75)	0.75	0.75 (0.13–4.19)
Bisexual	0.34	0.67 (0.22–2.04)	0.54	0.85 (0.19–1.98)
Son				
Yes	0.001	1.82 (1.63–2.09)	0.001	1.85 (1.68–2.21)
No	0.2	0.94 (0.84–1.57)	0.12	0.87 (0.75–1.10)
Residences				
North	0.002	1.50 (1.14–1.97)	0.004	1.63 (1.16–2.28)
Central	0.01	0.64 (0.48–0.84)	0.002	1.52 (1.14–2.37)
South and Islands	0.4	1.04 (0.78–1.38)	0.4	1.16 (0.81–1.65)
Education				
Primary school	0.34	1.12 (0.87–1.24)	0.49	1.22 (1.11–1.78)
Secondary school	0.45	1.20 (1.01–1.36)	0.67	1.32 (1.20–1.55)
High school	0.67	1.17 (1.08–1.45)	0.82	1.24 (1.14–1.37)
Graduate school	0.55	1.24 (1.15–1.57)	0.76	1.31 (1.20–1.68)
Occupation				
Student	0.06	1.67 (0.91–3.06)	0.08	1.57 (1.10–3.58)
Retired	0.06	0.27 (0.10–0.68)	0.5	0.8 (0.4–1.67)
Unemployed	0.02	1.62 (1.19–2.35)	0.04	1.45 (1.20–3.25)
Working at the usual workplace	0.39	1.04 (0.82–1.36)	0.4	0.75 (0.31–1.52)
Smart working	0.001	1.27 (1.10–1.68)	0.01	1.32 (1.15–1.76)
Married				
Yes	0.04	1.28 (0.98–1.67)	0.83	1.35 (1.10–1.57)
No	0.04	0.78 (0.59–1.01)	0.78	0.85 (0.64–1.21)
Years of stable relationships				
<5 years	0.04	0.77 (0.58–1.02)	0.7	0.94 (0.74–3.45)
≥5 years	0.04	1.29 (0.98–1.71)	0.02	1.49 (1.13–4.58)
Psychological data				
Anxiety	<0.0001	2.36 (1.60–3.48)	0.03	1.28 (0.78–2.09)
Tension	<0.0001	2.34 (1.64–3.34)	0.13	3.27 (2.51–5.34)
Fear	<0.0001	2.33 (1.63–3.30)	0.001	2.57 (1.78–4.16)
Insomnia	<0.0001	2.34 (1.64–3.34)	0.04	1.41 (0.94–2.13)

**Table 6:** Univariate analysis and multivariate logistic regression final model for male worsened sex lives vs. demographic and psychological data.

Male Worsening Sexual Life	Univariate Analysis		Logistic Regression	
	*p* Value	OR (95% CI)	*p* Value	OR (95% CI)
Age				
	0.74	0.67 (0.41–1.34)	0.89	0.78 (0.57–1.29)
BMI				
	0.59	0.58 (0.34–1.91)	0.66	0.61 (0.34–1.97)
Sexual Orientation				
Heterosexual	0.25	1.39 (0.45–4.26)	0.35	1.57 (0.74–4.23)
Homosexual	0.45	0.72 (0.38–1.98)	0.89	0.87 (0.34–5.23)
Bisexual	0.55	0.45 (0.27–2.59)	0.74	0.82 (0.32–2.65)
Son				
Yes	0.8	1.94 (1.54–2.23)	0.24	1.86 1.78–2.98)
No	0.8	0.92 (0.89–1.64)	0.24	0.97 (0.87–2.10)
Residences				
North	0	2.57 (1.25–2.58)	0.002	1.81 (1.24–2.59)
Central	0.7	0.86 (0.57–1.89)	0.003	1.48 (1.17–2.42)
South and Islands		1.44 (0.82–1.76)	0.35	1.78 (0.43–2.87)
Education				
Primary school	0.54	1.46 (0.91–3.24)	0.74	1.51 (1.23–2.14)
Secondary school	0.61	1.29 (1.17–1.47)	0.89	1.52 (1.20–2.18)
High school	0.85	1.28 (1.04–1.59)	0.94	2.14 (1.87–2.69)
Graduate school	0.45	1.78 (1.35–2.25)	0.87	1.54 (1.36–1.92)
Occupation				
Student	0.09	1.85 (1.25–3.78)	0.06	1.94 (1.20–4.58)
Retired	0.05	0.74 (0.21–0.84)	0.3	1.45 (1.15–1.82)
Unemployed	0.04	1.83 (1.49–2.75)	0.03	1.95 (1.54–3.25)
Working at the usual workplace	0.7	1.30 (0.47–2.41)	0.6	0.84 (0.45–1.78)
Smart working	0.01	1.57 (1.65–2.69)	0.02	3.24 (1.55–3.98)
Married				
Yes	0.02	1.36 (0.76–2.47)	0.74	1.98 (1.22–2.36)
No	0.02	0.95 (0.48–2.58)	0.54	0.93 (0.74–2.14)
Years of stable relationships				
<5years	0.03	0.48 (0.10–1.58)	0.4	1.24 (0.94–2.69)
≥5 years	0.03	1.78 (0.68–2.36)	0.01	2.36 (1.25–3.45)
Psychological data				
Anxiety	<0.0001	1.56 (0.45–2.87)	0.01	1.78 (0.92–3.45)
Tension	<0.0001	2.71 (1.36–3.56)	0.78	4.13 (2.63–6.21)
Fear	<0.0001	2.45 (1.79–4.56)	0.02	2.96 (1.61–4.57)
Insomnia	<0.0001	2.36 (1.87–4.51)	0.07	2.57 (1.45–3.68)

**Table 7:** Univariate analysis and multivariate logistic regression final model for female cohabitants worsening sex lives vs. demographic and psychological data.

Female Worsening Sexual Life	Univariate Analysis		Logistic Regression	
	*p* Value	OR (95% CI)	*p* Value	OR (95% CI)
Age				
	0.005	0.62 (0.45–0.86)	0.02	0.92 (0.65–1.23)
BMI				
	0.54	1.37 (1.01–1.79)	0.63	1.67 (1.35–1.86)
Sexual Orientation				
Heterosexual	0.25	1.85 (0.69–4.25)	0.34	1.93 (0.84–5.21)
Homosexual	0.36	0.75 (0.23–1.89)	0.87	0.84 (0.65–4.63)
Bisexual	0.65	0.94 (0.34–2.37)	0.78	0.61 (0.32–1.85)
Son				
Yes	0.51	1.29 (0.87–1.88)	0.26	1.74 (0.45–1.36)
No	0.32	0.66 (0.84–1.75)	0.26	0.82 (0.47–1.68)
Residences				
North	0.001	1.74 (1.35–2.35)	0.002	1.78 (1.45–2.80)
Central	0.03	0.53 (0.10–0.95)	0.001	1.92 (1.32–2.88)
South and Islands	0.5	1.26 (0.95–1.47)	0.6	1.45 (0.96–1.84)
Education				
Primary school	0.65	1.45 (0.95–1.63)	0.88	1.36 (1.14–1.95)
Secondary school	0.57	1.38 (1.10–1.78)	0.69	1.57 (1.32–1.86)
High school	0.82	1.26 (1.04–1.83)	0.71	1.49 (1.26–1.55)
Graduate school	0.63	1.87 (1.34–1.93)	0.86	1.61 (1.47–1.73)
Occupation				
Student	0.08	1.54 (0.84–3.54)	0.5	1.89 (1.30–3.84)
Retired	0.07	0.59 (0.16–0.84)	0.7	0.9 (0.12–1.75)
Unemployed	0.01	1.87 (1.36–2.73)	0.02	1.59 (1.25–3.59)
Working at the usual workplace	0.5	1.65 (0.69–1.95)	0.8	0.96 (0.47–1.73)
Smart working	0.002	1.43 (1.05–1.87)	0.01	1.63 (1.11–1.94)
Married				
Yes	0.03	0.94 (0.45–1.36)	0.91	0.75 (0.35–1.67)
No	0.03	1.45 (0.76–1.62)	0.84	1.61 (1.32–1.99)
Years of stable relationships				
<5 years	0.01	0.84 (0.41–1.57)	0.5	0.84 (0.54–3.75)
≥5 years	0.01	1.65 (0.86–1.92)	0.03	1.81 (1.35–4.98)
Psychological data				
Anxiety	<0.0001	2. 62 (1.72–4.11)	0.04	1.65 (0.52–2.79)
Tension	<0.0001	2.75 (1.75–4.80)	0.25	3.58 (2.47–5.88)
Fear	<0.014	1.01 (0.99–1.03)	0.02	2.67 (1.45–4.53)
Insomnia	<0.0001	2.26 (1.48–3.45)	0.01	1.78 (0.68–2.90)

## Data Availability

Not applicable.

## Supplementary Materials

The following are available online at https://www.mdpi.com/article/10.3390/jcm10071414/s1.
