# Supplementary material for: The Impact of Lockdown on Couples’ Sex Lives
Source: J Clin Med. 2021 Apr 1;10(7):1414. doi: 10.3390/jcm10071414 (PMC8037775; doi:10.3390/jcm10071414)
Supplement: Supplementary file 1 [file jcm-10-01414-s001.pdf]

Gentile sig./sig.ra,
Lo scopo di questa ricerca è quello di studiare l'impatto dell'emergenza sanitaria dovuta al COVID-19 sulle abitudini sessuali. Il progetto di ricerca è stato sviluppato dalla Prof.ssa Elisabetta Costantini Dipartimento di scienze Chirurgiche e biomediche - Università degli studi Di Perugia ed è in collaborazione con l'Università degli studi di Firenze, di Foggia, Bari, Verona, Roma Tor Vergata, Roma La Sapienza, Ospedale Monaldi di Napoli, Ospedale San Carlo di Nancy, Istituto clinica Zucchi, AO Bianchi Malacrino Morelli, Ospedale San Donato di Arezzo, Ospedale di Cuneo, AOU città della salute e della scienza di Torino, Ospedale San Lorenzo Carmagnola, Il responsabile della ricerca è la Prof. Elisabetta Costantini.

Cosa mi viene chiesto di fare? Le chiediamo di compilare la scheda anagrafica e rispondere ad alcune domande. I questionari riguardano la sessualità personale e di coppia durante il periodo di pandemia. Ogni questionario sarà preceduto da adeguate istruzioni che la aiuteranno a comprendere cosa fare. La partecipazione richiederà circa 10 minuti del suo tempo.
Il suo contributo sarà MOLTO utile per la ricerca e le sue possibili applicazioni. Grazie mille!

Informativa per il trattamento dei dati personali e sensibili a scopo di ricerca.

Disciplina della Privacy (art. 13 del D.Lgs. 196/2003)
*(*http://www.camera.it/parlam/leggi/deleghe/Testi/03196dl.htm*).*

Egregio Signore/a,

La informiamo che i dati raccolti saranno trattati in conformità al Decreto Legislativo 30 Giugno 2003, n. 196 "Codice in materia di protezione dei dati personali" che ha lo scopo di garantire il rispetto dei diritti, delle libertà fondamentali, nonché della dignità delle persone fisiche.

Per il trattamento di dati personali si intende qualunque operazione o complesso di operazioni svolte con o senza l'ausilio di mezzi elettronici o comunque automatizzati, concernenti la raccolta, la registrazione, l'organizzazione, la conservazione, l'elaborazione, la modificazione, la selezione, l'estrazione, il raffronto, l'utilizzo, l'interconnessione, il blocco, la comunicazione, la diffusione, la cancellazione e la distruzione dei dati stessi.

Ai sensi degli art. 7, 13 e 23 della predetta legge, chi effettua il trattamento di dati personali deve informare la persona cui i dati si riferiscono, specificando le finalità e modalità del trattamento, l'identità del responsabile del trattamento, dare notizia della natura obbligatoria o facoltativa del conferimento / assunzione dei dati, acquisire il consenso dell'interessato se richiesto.
I "dati sensibili", definiti dall'art.4, sono oggetto di particolare tutela, in quanto sono dati personali idonei a rilevare l'origine razziale ed etnica, le convinzioni religiose, filosofiche o di altro genere, le opinioni

politiche, l'adesione a partiti o sindacati, associazioni od organizzazioni a carattere religioso, filosofico, politico o sindacale, nonché dati personali idonei a rivelare lo stato di salute e la vita sessuale.
Tali dati possono essere trattati con il consenso scritto dell'interessato.
Le precisiamo inoltre quanto segue:
i dati che verranno raccolti in queste sessioni saranno utilizzati esclusivamente per lo studio di ricerca. Il risultato finale della ricerca, se valido, potrà essere oggetto di pubblicazione scientifica e sarà divulgato attraverso specifiche riviste del settore e potrà fornire supporto teorico agli esperti. Questa ricerca ha unicamente uno scopo scientifico e non sarà mai usata per scopi commerciali.
I risultati della ricerca non potranno essere diffusi se non nel rispetto dell'assoluto anonimato, pertanto i nomi dei partecipanti all'esperimento non saranno mai riportati in alcuna comunicazione.

Per contatti:
Prof.ssa Elisabetta Costantini
Email: elisabetta.costantini@unipg.it

Ai sensi dell'articolo 13 del D.L. 196/2003, la informiamo che i dati da lei forniti in forma anonima saranno trattati ai soli fini di ricerca.

CONSENSO INFORMATO

Essendo stato informato delle finalità dello studio io acconsento a compilare il questionario. Sono inoltre consapevole che, come previsto dal codice etico della Federazione Nazionale dei Medici Chirurghi ed Odontoiatri (http://www.fnomceo.it), i dati raccolti saranno archiviati in modo **completamente anonimo** e saranno trattati nel rispetto delle leggi vigenti in materia. I risultati della ricerca saranno utilizzati in forma anonima e unicamente per fini scientifici.
Posso ritirarmi dall'esperimento? In qualsiasi momento potrà interrompere il test senza dover dare alcuna giustificazione. Potrà inoltre decidere di ritirare il proprio consenso ed essere rimosso dallo studio in qualunque momento, scrivendo un messaggio all'indirizzo mail: **elisabetta.costantini@unipg.it**

Gentile utente,
sei stato invitato a compilare questa survey; ti preghiamo di rispondere alle domande successive, esclusivamente se:

- Non sei COVID positivo
- Se non sei single
- Se sei sessualmente attivo
- Se hai una relazione da più di 6 mesi

DATI GENERALI

Età

Sesso:

uomo

donna

Peso

Altezza

Orientamento sessuale:

eterosessuale

omossessuale

bisessuale

zona di residenza:

nord italia

centro italia

sud italia

isole

titolo di studio:

studi elementari

scuola media inferiore

scuola media superiore

laurea

occupazione:

disoccupato

studente

lavoratore attualmente in smart working

lavoratore presso il luogo di lavoro abituale

pensionato

hai figli:

si

no

numero di anni di relazione con il partner:

meno di 1 anno

da 1 anno a 3 anni

da 3 anni a 5 anni

maggiore 5 anni

convivi con il tuo partner

si

no

in caso di risposta negativa alla precedente domanda, durante il lockdown, hai avuto modo di avere contatti fisici con il tuo partner?

Si

no

"COVID 19 TEST"

Quanto si sente sicuro/a dentro casa?

- ☐ per niente
- ☐ poco
- ☐ mediamente
- ☐ molto
- ☐ moltissimo

Quanto si sente sicuro/a fuori casa?

- ☐ per niente
- ☐ poco
- ☐ mediamente
- ☐ molto
- ☐ moltissimo

Ritiene che la sua vita sessuale di coppia abbia subito un peggioramento in questo periodo?

- ☐ per niente
- ☐ poco
- ☐ mediamente
- ☐ molto
- ☐ moltissimo

Quanto si sente al sicuro in casa con il suo partner?

- ☐ per niente
- ☐ poco
- ☐ mediamente
- ☐ molto
- ☐ moltissimo

Ritiene che la sua vita sessuale di coppia abbia subito un miglioramento in questo periodo?

- ☐ per niente
- ☐ poco
- ☐ mediamente
- ☐ molto
- ☐ moltissimo

Quanto si sente al sicuro in casa con il suo partner?

- ☐ per niente
- ☐ poco
- ☐ mediamente
- ☐ molto
- ☐ moltissimo

Quanto si sente insoddisfatto in casa con il suo partner?

- ☐ per niente
- ☐ poco
- ☐ mediamente
- ☐ molto
- ☐ moltissimo
- ☐

Quanto si sente felice in casa con il suo partner?

- ☐ per niente
- ☐ poco
- ☐ mediamente
- ☐ molto
- ☐ moltissimo

Quanto si sente a disagio in casa con il suo partner?

- ☐ per niente
- ☐ poco
- ☐ mediamente
- ☐ molto
- ☐ moltissimo

Quanto si sente a suo agio in casa con il suo partner?

- ☐ per niente
- ☐ poco
- ☐ mediamente
- ☐ molto
- ☐ moltissimo

Quanto si sente soddisfatto in casa con il suo partner?

- ☐ per niente
- ☐ poco
- ☐ mediamente
- ☐ molto
- ☐ moltissimo

Ritiene che i suoi problemi di coppia siano diminuiti in questo periodo?

- ☐ per niente
- ☐ poco
- ☐ mediamente
- ☐ molto
- ☐ moltissimo

Quanto si sente infelice in casa con il suo partner?

- ☐ per niente
- ☐ poco
- ☐ mediamente
- ☐ molto
- ☐ moltissimo

Ritiene che i suoi problemi di coppia siano aumentati in questo periodo?

- ☐ per niente
- ☐ poco
- ☐ mediamente
- ☐ molto
- ☐ moltissimo

Si sente più nervosa/o nei confronti del suo partner in questo periodo?

- ☐ per niente
- ☐ poco
- ☐ mediamente
- ☐ molto
- ☐ moltissimo

Si sente più serena/o nei confronti del suo partner in questo periodo?

- ☐ per niente
- ☐ poco
- ☐ mediamente
- ☐ molto
- ☐ moltissimo

INTERNATIONAL INDEX OF ERECTILE FUNCTION

1) Nelle ultime quattro settimane quante volte è stato in grado di avere una erezione durante l'attività sessuale?

1. Non ho avuto alcuna attività sessuale
2. Quasi sempre o sempre
3. La maggior parte delle volte (molto più della metà delle volte)
4. Qualche volta (circa la metà delle volte)
5. Poche volte (molto meno della metà delle volte)
6. Quasi mai o mai

2) Nelle ultime 4 settimane quando ha avuto delle erezioni in seguito a stimolazione sessuale, quante volte erano sufficienti da permettere la penetrazione?

1. Non ho avuto alcuna attività sessuale
2. Quasi sempre o sempre
3. La maggior parte delle volte (molto più della metà delle volte)
4. Qualche volta (circa la metà delle volte)
5. Poche volte (molto meno della metà delle volte)
6. Quasi mai o mai

3) Nelle ultime 4 settimane, quando ha tentato di avere un rapporto sessuale, quante volte è stato in grado di penetrare la sua partner?

1. Non ho tentato di avere rapporti sessuali
2. Quasi sempre o sempre
3. La maggior parte delle volte (molto più della metà delle volte)
4. Qualche volta (circa la metà delle volte)
5. Poche volte (molto meno della metà delle volte)
6. Quasi mai o mai

4) Nelle ultime 4 settimane durante il rapporto sessuale, quanto spesso è stato in grado di mantenere l'erezione dopo aver penetrato la partner?

1. Non ho tentato di avere rapporti sessuali
2. Quasi sempre o sempre
3. La maggior parte delle volte (molto più della metà delle volte)
4. Qualche volta (circa la metà delle volte)
5. Poche volte (molto meno della metà delle volte)
6. Quasi mai o mai

5) Nelle ultime 4 settimane durante il rapporto sessuale quanto difficile è stato mantenere l'erezione fino al completamento del rapporto ?

1. Non ho tentato di avere rapporti sessuali
2. Estremamente difficile
3. Molto difficile
4. Difficile
5. Poco difficile
6. Per niente difficile

6) Nelle ultime 4 settimane quante volte ha tentato di avere rapporti sessuali?

1. Nessun tentativo

2. 1 – 2
3. 3 – 4
4. 5 – 6
5. 7 –10 tentativi
6. Più di 10 tentativi

7) Nelle ultime 4 settimane quando ha tentato di avere un rapporto sessuale, quanto spesso è stato soddisfacente per Lei Personalmente?

1. Non ho tentato di avere rapporti sessuali
2. Quasi sempre o sempre
3. La maggior parte delle volte (molto più della metà delle volte)
4. Qualche volta (circa la metà delle volte)
5. Poche volte (molto meno della metà delle volte)
6. Quasi mai o mai

8) Nelle ultime 4 settimane quanto piacevoli sono stati per lei i suoi rapporti sessuali?

1. Non ho avuto alcun un rapporto sessuale
2. Estremamente piacevoli
3. Molto piacevoli
4. Abbastanza piacevoli
5. Non molto piacevoli
6. Per niente piacevoli

9) Nelle ultime 4 settimane quando ha avuto una stimolazione oppure un rapporto sessuale, quanto ha eiaculato?

1. Non ho tentato di avere rapporti sessuali
2. Quasi sempre o sempre
3. La maggior parte delle volte (molto più della metà delle volte)
4. Qualche volta (circa la metà delle volte)
5. Poche volte (molto meno della metà delle volte)
6. Quasi mai o mai

10) Nelle ultime quattro settimane quando ha avuto una stimolazione sessuale oppure un rapporto sessuale, quanto spesso ha provato la sensazione d'orgasmo con o senza eiaculazione?

1. Non ho tentato di avere rapporti sessuali
2. Quasi sempre o sempre
3. La maggior parte delle volte (molto più della metà delle volte)
4. Qualche volta (circa la metà delle volte)
5. Poche volte (molto meno della metà delle volte)
6. Quasi mai o mai

tentativi tentativi tentativi

11) Nelle ultime 4 settimane quanto spesso ha provato desiderio sessuale?

1. Quasi sempre o sempre
2. Spesso(per la maggior parte del tempo)
3. Qualche volta(per buona parte del tempo)
4. Poche volte(per una piccola parte del tempo

5. Quasi mai o mai

12) Come valuterebbe il suo livello di desiderio sessuale relativo alle ultime 4 settimane?

1. Molto alto
2. Alto
3. Moderato
4. Basso
5. Molto basso o del tutto nullo

13) Nelle ultime 4 settimane in che misura è stato soddisfatto delle sua relazione sessuale con la partner?

1. Molto soddisfatto
2. Moderatamente soddisfatto
3. Più o meno ugualmente soddisfatto e insoddisfatto
4. Moderatamente insoddisfatto
5. Molto insoddisfatto

14) Nelle ultime 4 settimane in che misura è stato soddisfatto della sua relazione sessuale con la partner?

1. Molto soddisfatto
2. Moderatamente soddisfatto
3. Più o meno ugualmente soddisfatto e insoddisfatto
4. Moderatamente insoddisfatto
5. Molto insoddisfatto

15) Nelle ultime 4 settimane come valuterebbe il suo livello di fiducia nel poter raggiungere e mantenere una erezione?

1. Molto alto
2. Alto
3. Moderato
4. Basso
5. Molto basso o del tutto nullo

FEMALE SEXUAL FUNCTION INDEX

1 - Nell'arco delle precedenti 4 settimane, quanto di sovente ha avuto desiderio od interesse sessuale?

Quasi sempre o sempre
La maggior parte delle volte (più della metà del tempo)
Delle volte (circa la metà del tempo)
Alcune volte (meno della metà delle volte)
Quasi mai o mai

2 - Nell'arco delle precedenti 4 settimane, come valuterebbe il suo livello (grado) di desiderio od interesse sessuale?

Molto alto
Alto
Moderato
Basso
Molto basso od assente

3 - Nell'arco delle precedenti 4 settimane, quanto di sovente ha percepito piacere durante l'attività od il rapporto sessuale?

Nessuna attività sessuale

Quasi sempre o sempre

La maggior parte delle volte (più della metà)

Qualche volta (circa la metà)

Poche volte (meno della metà)

Quasi mai o mai

4 - Nell'arco delle precedenti 4 settimane, come quantificherebbe il suo livello di piacere durante l'attività od il rapporto sessuale?

Nessuna attività sessuale

Molto alto

Alto

Moderato

Basso

Molto basso od assente

5 - Nell'arco delle precedenti 4 settimane, quanto si è sentita predisposta a provare piacere durante l'attività od il rapporto sessuale?

Nessuna attività sessuale

Molto predisposta

Predisposta

Moderatamente predisposta

Poco predisposta

Poco o per nulla predisposta

6 - Nell'arco delle precedenti 4 settimane, quanto di sovente si è ritenuta soddisfatta del suo piacere (eccitazione) durante l'attività od il rapporto sessuale?

Nessuna attività sessuale

Quasi sempre o sempre

La maggior parte delle volte (più della metà)

Qualche volta (circa la metà)

Poche volte (meno della metà)
Quasi mai o mai

7 - Nell'arco delle precedenti 4 settimane, quanto di sovente si è sentita lubrificata ("bagnata") durante l'attività od il rapporto sessuale?

Nessuna attività sessuale
Quasi sempre o sempre
La maggior parte delle volte (più della metà)
Qualche volta (circa la metà)
Poche volte (meno della metà)
Quasi mai o mai

8 - Nell'arco delle precedenti 4 settimane, quanto di sovente ha avuto difficoltà a essere lubrificata ("bagnata") durante l'attività od il rapporto sessuale?

Nessuna attività sessuale
Estremamente difficoltosa o impossibile
Molto difficoltosa
Difficoltosa
Lievemente difficoltosa
Non difficoltosa

9 - Nell'arco delle precedenti 4 settimane, quanto di sovente ha mantenuto la lubrificazione sino alla fine dell'attività od il rapporto sessuale?

Nessuna attività sessuale
Quasi sempre o sempre
La maggior parte delle volte (più della metà)
Qualche volta (circa la metà)
Poche volte (meno della metà)
Quasi mai o mai

10 - Nell’arco delle precedenti 4 settimane, quanto di sovente ha avuto difficoltà a mantenere la lubrificazione sino alla fine dell’attività od il rapporto sessuale?

Nessuna attività sessuale
Estremamente difficoltosa o impossibile
Molto difficoltosa
Difficoltosa
Lievemente difficoltosa
Non difficoltosa

11 - Nell’arco delle precedenti 4 settimane, quando ha avuto attività od il rapporto sessuale, quanto di sovente ha raggiunto l’orgasmo?

Nessuna attività sessuale
Quasi sempre o sempre
La maggior parte delle volte (più della metà)
Qualche volta (circa la metà)
Poche volte (meno della metà)
Quasi mai o mai

12 - Nell’arco delle precedenti 4 settimane, quando ha avuto attività od il rapporto sessuale, quanto difficoltoso è stato raggiungere l’orgasmo?

Nessuna attività sessuale
Estremamente difficoltosa o impossibile
Molto difficoltosa
Difficoltosa
Lievemente difficoltosa
Non difficoltosa

13 - Nell'arco delle precedenti 4 settimane quanto si è ritenuta soddisfatta della sua abilità nel raggiungere l'orgasmo?

Nessuna attività sessuale
Molto soddisfatta
Moderatamente soddisfatta
Più o meno soddisfatta
Moderatamente insoddisfatta
Non soddisfatta

14 - Nell'arco delle precedenti 4 settimane quanto si è ritenuta soddisfatta del grado d'intimità con il suo partner durante l'attività od il rapporto sessuale?

Nessuna attività sessuale
Molto soddisfatta
Moderatamente soddisfatta
Più o meno soddisfatta
Moderatamente insoddisfatta
Non soddisfatta

15 - Nell'arco delle precedenti 4 settimane quanto si è ritenuta soddisfatta dell'intesa sessuale con il suo partner durante l'attività od il rapporto sessuale?

Nessuna attività sessuale
Molto soddisfatta
Moderatamente soddisfatta
Più o meno soddisfatta
Moderatamente insoddisfatta
Non soddisfatta

16 - Nell'arco delle precedenti 4 settimane quanto si è ritenuta soddisfatta in generale della sua vita sessuale?

Nessuna attività sessuale
Molto soddisfatta
Moderatamente soddisfatta
Più o meno soddisfatta
Moderatamente insoddisfatta
Non soddisfatta

17 - Nell'arco delle precedenti 4 settimane, quanto di sovente ha avuto dolore durante la penetrazione vaginale?

Nessuna penetrazione vaginale
Quasi sempre o sempre
La maggior parte delle volte (più della metà)
Qualche volta (circa la metà)
Poche volte (meno della metà)
Quasi mai o mai

18 - Nell'arco delle precedenti 4 settimane, quanto di sovente ha avuto dolore dopo la penetrazione vaginale?

Nessuna penetrazione vaginale
Quasi sempre o sempre
La maggior parte delle volte (più della metà)
Qualche volta (circa la metà)
Poche volte (meno della metà)
Quasi mai o mai

19 - Nell'arco delle precedenti 4 settimane, come quantificherebbe il suo fastidio o dolore durante e dopo la penetrazione vaginale?

Nessuna penetrazione vaginale
Nolto alto

Alto
Moderato
Basso
Molto basso o assente

HAM-A: Hamilton Anxiety Rating Scale (Scala di Hamilton della Valutazione dell'Ansia)

Di seguito è riportato un elenco di frasi che descrivono delle sensazioni che hanno le persone. Compilate il questionario con la risposta che meglio descrive la misura in cui si hanno le seguenti condizioni.

Stato d'animo ansioso Preoccupazioni, anticipazione del peggio, anticipazioni timorose, irritabilità.
0 = Non presente, 1 = Leggero, 2 = Moderato, 3 = Grave, 4 = Molto grave
Tensione Sentimenti di tensione, affaticabilità, risposte di allarme, commozione fino alle lacrime, tremore, sensazione di irrequietezza, incapacità di rilassarsi.
0 = Non presente, 1 = Leggero, 2 = Moderato, 3 = Grave, 4 = Molto grave
Paure Del buio, degli stranieri, di essere lasciati soli, di animali, del traffico, delle folle.
0 = Non presente, 1 = Leggero, 2 = Moderato, 3 = Grave, 4 = Molto grave
Insonnia Difficoltà ad addormentarsi, sonno interrotto, sonno insoddisfacente e stanchezza al risveglio, sogni, incubi, terrori notturni.
0 = Non presente, 1 = Leggero, 2 = Moderato, 3 = Grave, 4 = Molto grave
Intellettuale Difficoltà di concentrazione, scarsa memoria.
0 = Non presente, 1 = Leggero, 2 = Moderato, 3 = Grave, 4 = Molto grave
Stato d'animo depresso Perdita di interesse, mancanza di piacere a hobby, depressione, risveglio precoce, altalena diurno.
0 = Non presente, 1 = Leggero, 2 = Moderato, 3 = Grave, 4 = Molto grave
Livello somatico (muscolare) Dolori e sofferenze, spasmi, rigidità, scatti mioclonici, digrignamento di denti, voce incerta, aumento del tono muscolare.
0 = Non presente, 1 = Leggero, 2 = Moderato, 3 = Grave, 4 = Molto grave 8.
Livello somatico (sensoriale) Tinnito, offuscamento della vista, vampate calde e fredde, sensazione di debolezza, sensazione di punture.
0 = Non presente, 1 = Leggero, 2 = Moderato, 3 = Grave, 4 = Molto grave

Sintomi cardiovascolari Tachicardia, palpitazioni, dolore al petto, palpitazione di vasi, sensazioni di svenimento, mancanza ritmo.

0 = Non presente, 1 = Leggero, 2 = Moderato, 3 = Grave, 4 = Molto grave

Sintomi respiratori Pressione o costrizione al petto, sensazione di soffocamento, sospiri, dispnea. 0 = Non presente, 1 = Leggero, 2 = Moderato, 3 = Grave, 4 = Molto grave

Sintomi gastrointestinali Difficoltà a deglutire, dolori addominali, sensazione di bruciore, pienezza addominale, nausea, vomito, borborigmi, scioltezza delle viscere, perdita di peso, stipsi.

0 = Non presente, 1 = Leggero, 2 = Moderato, 3 = Grave, 4 = Molto grave

Sintomi urogenitali Frequenza della minzione, urgenza della minzione, amenorrea, menorragia, sviluppo di frigidità, eiaculazione precoce, perdita di libido, impotenza.

0 = Non presente, 1 = Leggero, 2 = Moderato, 3 = Grave, 4 = Molto grave

Sintomi autonomi Secchezza delle fauci, vampate di calore, pallore, tendenza alla sudorazione, vertigini, tensione, cefalea, caduta di capelli.

0 = Non presente, 1 = Leggero, 2 = Moderato, 3 = Grave, 4 = Molto grave

Sei sposato?

Si.

No

MARITAL ADJUSTMENT TEST (MAT)

Quando sorgono dei disaccordi, di solito risultano in:

- in marito che cede
- in moglie che cede
- in accordo con reciproco dare e avere

Lei e il suo compagno avete degli interessi insieme?

- Tutti
- alcuni
- pochi
- nessuno

Nel tempo libero generalmente preferisci:

- Andare fuori
- Stare a casa

Il tuo compagno generalmente preferisce:

- Andare fuori
- Stare a casa

Hai mai desiderato di non esservi sposati?

- Frequentemente
- Occasionalmente
- Raramente
- mai

Se avessi la tua vita da rivivere, pensi che

- Sposeresti la stessa persona
- Sposeresti una altra persona
- Non ti sposeresti affatto.

Ti confidi con il tuo compagno:

- Mai
- Raramente
- spesso
- Sempre
